# Travelling-Wave and Asymptotic Analysis of a Multiphase Moving Boundary Model for Engineered Tissue Growth

**DOI:** 10.1007/s11538-022-01044-0

**Published:** 2022-07-12

**Authors:** Jacob M. Jepson, Nabil T. Fadai, Reuben D. O’Dea

**Affiliations:** School of Mathematical Sciences, University of Nottingham, Nottingham, NG7 2RD UK

**Keywords:** Tissue engineering, Reaction–diffusion model, Darcy’s law

## Abstract

We derive a multiphase, moving boundary model to represent the development of tissue in vitro in a porous tissue engineering scaffold. We consider a cell, extra-cellular liquid and a rigid scaffold phase, and adopt Darcy’s law to relate the velocity of the cell and liquid phases to their respective pressures. Cell–cell and cell–scaffold interactions which can drive cellular motion are accounted for by utilising relevant constitutive assumptions for the pressure in the cell phase. We reduce the model to a nonlinear reaction–diffusion equation for the cell phase, coupled to a moving boundary condition for the tissue edge, the diffusivity being dependent on the cell and scaffold volume fractions, cell and liquid viscosities and parameters that relate to cellular motion. Numerical simulations reveal that the reduced model admits three regimes for the evolution of the tissue edge at large time: linear, logarithmic and stationary. Employing travelling-wave and asymptotic analysis, we characterise these regimes in terms of parameters related to cellular production and motion. The results of our investigation allow us to suggest optimal values for the governing parameters, so as to stimulate tissue growth in an engineering scaffold.

## Introduction

In vitro tissue engineering is a form of regenerative medicine which often involves seeding cells into a porous bio-engineered scaffold that allows nutrient transport, structural support and a means for cell signalling activity (Chan and Leong 2008). Subject to the correct environment and growth factors, the cells will develop into a functional construct that can be used to restore damaged tissues and improve concepts in pharmaceutical research such as experimental drug therapy (Jensen et al. 2018). Employing contributions from an assortment of scientific fields, tissue engineering is considered an interdisciplinary practice that has the potential to benefit a substantial proportion of the global population with devastating soft tissue, bone and whole organ diseases (Dzobo et al. 2018). The field of tissue engineering has enjoyed many successes; for example, the generation, replacement and longevity of engineered bones and bronchial tubes derived from the recipients’ cells (Sato et al. 2008; Petite et al. 2000; Schimming and Schmelzeisen 2010). However, a shortage in the supply of donor tissue creates a demand on the field to make engineered tissue routinely clinically available (Levitt 2015). Whilst the field is rich in both theoretical and experimental knowledge, a lack of understanding regarding the processes by which cells assemble into tissues means that viable replacement constructs are only available in a minority of cases.

Extensive mathematical research has been undertaken to make sense of the complicated mechanisms within tissue growth. Some authors adopt a microscale approach, which can take the form of cellular automaton systems (Vitvitsky 2014; Lehotzky and Zupanc 2019; Youssef 2015) that seek to model interactions between a large number of individual cells. Whilst such systems can track the behaviour of cells, they can become computationally infeasible for tissue-scale simulations (Ermentrout and Edelstein-Keshet 1993). Some authors adopt a probabilistic approach (Fadai et al. 2019; Browning et al. 2019a, b) and exploit experimental data to explore ways in which tissue engineering techniques can be improved. For example, Sogutlu and Koc (2013) present a stochastic model to determine the expected required number of pores for each region of a tissue engineering scaffold.

Conversely, continuum models (O’Dea et al. 2010; Lemon et al. 2006; Breward et al. 2002; Eyles et al. 2019; Byrne et al. 2003 ) track the evolution of tissue constituents by employing systems of partial differential equations. Whilst continuum models cannot impose rules on individual cells, they can be derived by imposing averaging techniques (Drew 1983) on equations that govern cellular behaviour at a microscopic level, from which relevant mathematical techniques may be exploited to determine relationships between parameters and model behaviours. The study of in vitro tissue growth via continuum models is extensive, see O’Dea et al. (2012) and Klika et al. (2016) for reviews; of particular relevance to this study, however, Lemon et al. (2006) considers a continuum multiphase model to investigate how mechanical pressures within growing tissue influence the aggregation or dispersion of cells in a scaffold, and relates the existence of these regimes to the governing parameters. Lemon and King (2007a) examines travelling-wave solutions of the multiphase model formulated in Lemon et al. (2006) and find that in certain limits, the tissue propagates through the scaffold at a constant speed as either a forward or backward travelling wave, dependent on parameter values.

In this paper, we develop and analyse a continuum multiphase model that represents the development of tissue in vitro in an artificial scaffold. In our model, we aim to capture key features of tissue growth and extinction whilst developing a tractable formulation. In particular, we consider a porous flow description comprising a tissue cell phase, extra-cellular liquid phase and a scaffold phase, the former two being modelled as incompressible fluids and the latter as an inert solid. Tissue mechanics are accounted for by considering relevant constitutive assumptions in a similar fashion to those presented in Lemon et al. (2006) and Lemon and King (2007a). The model is reduced to a reaction–diffusion equation for the cell phase and a moving boundary condition for the tissue edge, after which travelling-wave, asymptotic and numerical methods are employed to deduce the resulting solution behaviour. The paper is constructed as follows. In Sect. 2, we formulate and subsequently reduce and non-dimensionalise the model. In Sect. 3, we present and discuss numerical solutions to the reduced model, which motivate the travelling-wave and asymptotic analyses conducted in Sects. 4 and 5. In Sect. 6, we draw some conclusions regarding the behaviour of the model and interpret the mathematical results in terms of the biological application.

## Model Development

We construct a multiphase model to describe the growth of a nutrient rich tissue within a porous tissue engineering scaffold. For simplicity, we formulate the model in a one-dimensional Cartesian geometry. The model consists of three phases: two of which are fluid phases denoted by $n(x,\,t)$ and $w(x,\,t),$ and represent the volume fraction of cells and extra-cellular liquid, respectively. A rigid, non-degradable scaffold with uniform volume fraction *s* is the third phase and remains constant, the porosity of the scaffold hence being given by $1-s.$ Cell growth and death occur via mass transfer between *n* and *w*. The phases satisfy the no voids volume constraint:

1 $$\begin{aligned} n+w+s=1. \end{aligned}$$

The velocity fields $v_n(x,\,t)$ and $v_w(x,\,t),$ and pressures $p_n(x,\,t)$ and $p_w(x,\,t),$ are associated with the phases *n* and *w* accordingly. The spatial domain of the tissue evolves over time due to cellular motion, so we track it with a moving boundary, $x=L(t).$ In the subsections that follow, we state equations that govern mass transfer between *n* and *w*,  as well as provide constitutive assumptions for $v_n,\,v_w,\,p_n$ and $p_w$ suitable to describe tissue growth in a scaffold. We state necessary initial and boundary conditions for the variables and the moving boundary *L*(*t*), and simplify and non-dimensionalise the model.

### Governing Equations

We assume that cells proliferate and assemble daughter cells from the available liquid and that when cells die, they decompose and dissolve into the liquid phase. In view of these processes, it is reasonable to follow Lemon et al. (2006), Byrne et al. (2003), Breward et al. (2002) and Preziosi and Tosin (2003) (and many others) and assume the densities of *n* and *w* to be equal. Following these assumptions, the mass transfer equations can be represented as

2 $$\begin{aligned} \frac{\partial n}{\partial t}+\frac{\partial }{\partial x}(nv_n) = \varGamma (n,\,w) \quad \quad \text {and} \quad \quad \frac{\partial w}{\partial t}+\frac{\partial }{\partial x}(wv_w) = -\varGamma (n,\,w), \end{aligned}$$

where $\varGamma$ is the net rate of cell proliferation. Adding the equations from (2) results in the overall conservation of mass condition

3 $$\begin{aligned} \frac{\partial }{\partial x}[nv_n+(1-n-s)v_w]=0, \end{aligned}$$

where (1) has been used to eliminate the time derivative and to replace *w* with $1-n-s.$

Noting that *n* and *w* are modelled as fluids and *s* as a porous scaffold, we take the interphase drags to be dominated by those with the scaffold and neglect that between the tissue and liquid. In view of this, we apply Darcy’s law to relate the velocity of the cell and liquid phases to their respective pressures. Following King and Franks (2004) and Eyles et al. (2019), we take

4 $$\begin{aligned} v_n = -\frac{K}{\mu _n(n,w)}\frac{\partial p_n }{\partial x} \quad \quad \text {and} \quad \quad v_w = -\frac{K}{\mu _w(n,w)}\frac{\partial p_w }{\partial x}, \end{aligned}$$

where $\mu _n$ and $\mu _w$ represent the viscosity of the cell and liquid phases and *K* is the permeability of the scaffold.

Remaining consistent with Lemon et al. (2006) and Lemon and King (2007a, 2007b), we relate the cellular and extra-cellular liquid pressures via

5 $$\begin{aligned} p_n = p_w+\Sigma (n,\,s), \end{aligned}$$

where $\Sigma$ represents extra pressures that arise due to cell–cell and cell–scaffold interactions. Since the scaffold is assumed to be inert and of uniform porosity, we suppress the dependence $\Sigma$ has on *s* from hereon for brevity. We note that combining (5) with the relations from (4) allows the elimination of $p_n$ and $p_w$ and provides

6 $$\begin{aligned} v_n = \frac{\mu _w}{\mu _n}v_w-\frac{K}{\mu _n}\frac{\partial \Sigma }{\partial x}. \end{aligned}$$

### Initial and Boundary Conditions

Assuming the tissue to be symmetric about its centre ($x=0$), we take

7 $$\begin{aligned} v_n(x,\,t) = v_w(x,\,t) = 0 \quad \quad \text {at} \quad \quad x=0. \end{aligned}$$

Naturally, the cell volume fraction is identically zero at the edge of the tissue:

8 $$\begin{aligned} n(x,\,t)=0 \quad \quad \text {at} \quad \quad x=L(t). \end{aligned}$$

The moving boundary *L*(*t*) moves with the cell velocity, hence

9 $$\begin{aligned} \frac{\mathrm {d}L(t)}{\mathrm {d} t} = v_n( L(t),\,t). \end{aligned}$$

The initial distribution of *n* and tissue boundary position, respectively, are denoted by

10 $$\begin{aligned} n(x,\,0) = n_0(x) \quad \quad \text {and} \quad \quad L(0)=L_0. \end{aligned}$$

### Model Reduction

We reduce the model to a reaction–diffusion equation and a moving boundary condition. Integrating (3) and applying the boundary conditions from (7) provides

11 $$\begin{aligned} v_n = -\varPhi (n)\frac{\partial n}{\partial x}, \quad \quad \text {where} \quad \quad \varPhi = \frac{K(1-n-s)}{\mu _n(1-n-s)+\mu _w n}\frac{\mathrm {d}\Sigma }{\mathrm {d}n}. \end{aligned}$$

Here, we note $\mu _n$ and $\mu _w$ are assumed to be independent of *n* for simplicity. Substituting (11) into the first of (2) provides the reaction–diffusion equation:

12 $$\begin{aligned} \frac{\partial n}{\partial t}=\frac{\partial }{\partial x}\bigg (n\varPhi (n)\frac{\partial n}{\partial x}\bigg )+\varGamma (n,\,w). \end{aligned}$$

Combining (9) with (11) provides the moving boundary condition:

13 $$\begin{aligned} \frac{\mathrm {d}L}{\mathrm {d}t}=-\varPhi (0)\frac{\partial n}{\partial x}(L(t),\,t), \end{aligned}$$

where the boundary condition from (8) provides $\varPhi (n)=\varPhi (0)$ at $x=L(t).$ Finally, (11) implies the boundary condition on $v_n$ from (7) becomes

14 $$\begin{aligned} \varPhi (n)\frac{\partial n}{\partial x}=0 \quad \quad \text {at} \quad \quad x=0. \end{aligned}$$

### Constitutive Assumptions

We now define constitutive assumptions for $\varGamma$ and $\Sigma$ that are suitable to describe tissue growth in a rigid scaffold. We assume that daughter cells are constructed via mitosis using the available liquid and that when cells die via apoptosis, they dissolve into the liquid. Thus, we take

15 $$\begin{aligned} \varGamma (n)= r_mn(1-n-s)-r_an, \end{aligned}$$

where $r_m$ and $r_a$ are the positive constant rates of cell mitosis and apoptosis, and (1) is used to replace *w* with $1-n-s.$ Following Lemon et al. (2006) and Lemon and King (2007a, 2007b), an appropriate expression for $\Sigma (n)$ is

16 $$\begin{aligned} \Sigma (n) = \underbrace{ \frac{\delta _n n^2}{(1-n-s)}+\nu n}_{\text {cell--cell interactions}}+ \underbrace{\frac{\delta _s s n}{(1-n-s)}-\chi s}_{\text {cell--scaffold interactions}}, \end{aligned}$$

for $\nu \in \mathbb {R}$ and positive constants $\delta _n,\,\delta _s$ and $\chi .$ The first term in (16) represents repulsive forces exerted between the cells at high volume fractions, as characterised by the singularity at $n=1-s.$ The second term represents the propensity for cells to disperse or aggregate, with $\nu$ taking a positive or negative value accordingly. The third term represents repulsive forces that occur due to cell–scaffold interactions, whilst the fourth describes attractive forces between the cells and scaffold. For simplicity, we take $\delta :=\delta _n=\delta _s$. We note that $\varPhi (n)$ must be strictly positive to prevent negative diffusion in (12) and nonlinear degeneracy in (13). This is achieved when $\nu >0,$ which is henceforth assumed. Physically, this corresponds to a tendency for cells to spread through the scaffold (Lemon et al. 2006).

### Non-dimensionalisation and Parameter Values

We non-dimensionalise (12), (13) and the initial and boundary conditions from (8),  (10) and (14). By introducing the dimensionless variables

17 $$\begin{aligned} \widehat{t} = r_m t, \qquad \qquad \widehat{x}= \sqrt{\frac{r_m}{\varPhi (0)}}x, \qquad \qquad \widehat{L}= \sqrt{\frac{r_m}{\varPhi (0)}}L, \end{aligned}$$

the following dimensionless model results:

18 $$\begin{aligned}&\frac{\partial n}{\partial \widehat{t}}=\frac{\partial }{\partial \widehat{ x}}\bigg (n\phi (n)\frac{\partial n}{\partial \widehat{x}}\bigg )+n(\kappa -n), \qquad 0<\widehat{x}<\widehat{L}, \end{aligned}$$

19 $$\begin{aligned}&\frac{\mathrm {d}\widehat{L}}{\mathrm {d}\widehat{t}}=-\frac{\partial n}{\partial \widehat{x}}(\widehat{L},\,\widehat{t}\,), \end{aligned}$$

20 $$\begin{aligned}&\phi (n)\frac{\partial n}{\partial \widehat{x}}\bigg |_{\widehat{x}=0}=0, \quad \quad n(\widehat{L},\,\widehat{t}\,)=0, \quad \quad \widehat{L}(0)=\widehat{L}_0, \quad \quad n(\widehat{x},\,0) = n_0(\widehat{x}), \end{aligned}$$

where $\kappa = 1-s-r_a/r_m$ and $\widehat{L}_0=L_0\sqrt{r_m/\varPhi (0)}$. We also have

21 $$\begin{aligned} \phi (n) = \frac{\varPhi (n)}{\varPhi (0)}= \frac{(1-s)(1-n-s)}{(\eta (1-s)+s)(1-(\mu -1)n-s)}\bigg [\frac{(1-s)}{(1-n-s)^2}+\eta -1\bigg ] \end{aligned}$$

where $\eta =\nu /\delta$ and $\mu =\mu _w/\mu _n.$ In the proceeding, we dispense of the hat notation for clarity.

The parameter $\kappa$ is shown in subsequent sections to be of crucial importance to the qualitative features of the model solutions. Physically, $\kappa$ represents the difference between the scaffold porosity and the ratio between the cell death and growth rates. We note that the scaffold permeability parameter *K*,  as seen in (11) is not present in the non-dimensional model (18)–(20). However, the scalings selected in (17) imply that the dimensional tissue boundary position increases with the scaffold permeability. A linear stability analysis around the steady states of (18), $n=0$ and $n=\kappa$, is conducted in “Appendix A” and shows that the former is stable when $\kappa <0$ and the latter when $\kappa >0.$ In view of this, we are primarily motivated to investigate (18)–(20) for different values of $\kappa ,$ though variations in $s,\,\mu ,$ and $\eta$ will also be considered in part so as to deduce their optimal values for the stimulation of tissue growth. Unless otherwise stated, we take $\mu =\eta =1$ and we adopt the initial conditions

22 $$\begin{aligned} n_0(x) = \omega (1-x^2) \quad \quad \text {and} \quad \quad L_0=1, \end{aligned}$$

so that $\omega$ denotes the cell volume fraction at $x=0.$ Following Lemon and King (2007a, 2007b), and unless otherwise stated, we set $s=0.2$ and $\omega =0.03,$ the former corresponding to a scaffold with a porosity of 0.8 and is consistent with the experimental study presented in Malda et al. (2004).

## Numerical Results

We present and discuss the numerical solutions for $n(x,\,t)$ and *L*(*t*) from the PDE system (18)–(20), paying separate attention to the cases $\kappa >0,\,\kappa <0$ and $\kappa =0.$ For numerical convenience, we fix the moving boundary by introducing the variable transform $\xi =x/L(t)$ so that $\xi \in [0,\,1],$ which means (18) and (19) become

23 $$\begin{aligned}&\frac{\partial n}{\partial t}=\frac{\xi }{L}\frac{\mathrm {d}L}{\mathrm {d}t}\frac{\partial n}{\partial \xi }+\frac{1}{L^2}\frac{\partial }{\partial \xi }\bigg (n\phi (n)\frac{\partial n}{\partial \xi }\bigg )+n(\kappa -n), \end{aligned}$$

24 $$\begin{aligned}&\frac{\mathrm {d}L}{\mathrm {d}t}=-\frac{1}{L} \frac{\partial n}{\partial \xi }(1,\,t). \end{aligned}$$

Subject to the transformed boundary and initial conditions from (20), we numerically integrate (23) and (24) by discretising first- and second-order spatial derivatives using second-order finite differences. Upwind finite differences were used for the second term of (23). Temporal derivatives are numerically integrated by utilising ode23s in MATLAB.

**Fig. 1:**
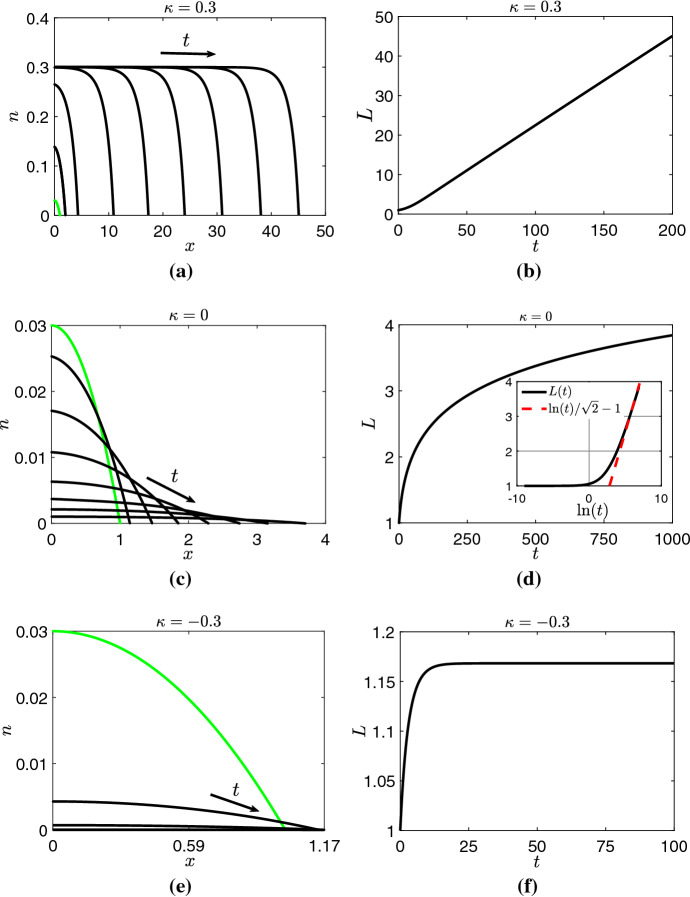
Numerical solutions of the system of PDEs from (18)–(20), with **a**, **c**, **e** showing $n(x,\,t)$ and **b**, **d**, **f** showing *L*(*t*) for different values of $\kappa$. The green lines in **a**, **c**, **e** represent the initial conditions $n_0(x)$ from (22), whereas the black lines represent *n* for different values of *t*. The black arrows point in the direction of increasing time. The dashed red line in **d** is given by $\ln (t)/\sqrt{2}-1$ and highlights the logarithmic growth of *L* at large time. Parameter values: $L_0=\mu =\eta =1,\,\omega =0.03$ and $s=0.2$ (Color figure online)

For $\kappa =0.3$, as shown in Fig. 1a, b, we observe semi-infinite travelling waves in *n* and linear growth in *L* after a period of transient growth from their initial states. For $\kappa =0$, as shown in Fig. 1c, we observe *n* decaying from the initial data. Figure 1d shows unbounded growth in *L*. The inset shows *L*(*t*) and the function $\ln (t)/\sqrt{2}-1$ plotted against $\ln (t)$, from which we conclude that *L* grows logarithmically at large time. For $\kappa =-0.3$, as shown in Fig. 1e, we observe that *n* decays from the initial data more quickly than for $\kappa =0$. The initial growth of *L* shown in Fig. 1f occurs due to the diffusion of *n* from the initial state; however, we observe the eventual formation of a steady state. Numerical simulations that are not included here suggest that travelling-wave and steady-state behaviour is exhibited by (18)–(20) for all $\kappa >0$ and $\kappa <0$ accordingly.

Clearly, the case in which $\kappa >0$ corresponds to effective tissue growth. This motivates a travelling-wave analysis of (18)–(20) for $\kappa >0$ which is presented in Sect. 4, where we express the speed of the tissue edge in terms of the governing parameters. In Sect. 5, asymptotic solutions for *n* and *L* are found when $0<\kappa \ll 1,$ so that the cell distribution and tissue speed are explicitly available. Whilst the case $\kappa <0$ results in tissue decay, an asymptotic analysis of (18)–(20) for this case is presented in Sect. 5. Overall, the results in this section suggest that $\kappa >0$ must hold for tissue growth to occur, thus suggesting that tissue engineers should ensure that the porosity of the scaffold is at least larger than the ratio between the rate of cell death and growth.

## Travelling-Wave Analysis for $\kappa >0$

Figure 1a, b indicates the emergence of semi-infinite travelling waves of constant speed for $\kappa >0.$ In light of this, we assume that for sufficiently large time, $L\sim ct$ where *c* is the constant wave speed at which the tissue edge moves. In this section, we employ travelling-wave analysis to obtain the wave speed *c* in terms of the governing parameters when $\kappa >0$.

We transform (18)–(20) via the travelling-wave coordinates $z = x-L\sim x-ct$ where $z \in (-\infty ,\,0]$. Setting $n(x-ct)=n(z)$, we obtain

25 $$\begin{aligned} -c\frac{\mathrm {d}n}{\mathrm {d}z}=\frac{\mathrm {d}}{\mathrm {d}z}\bigg (n\phi (n)\frac{\mathrm {d}n}{\mathrm {d}z}\bigg )+n(\kappa -n), \end{aligned}$$

26 $$\begin{aligned} n(0)=0, \quad \quad c = -\frac{\mathrm {d}n}{\mathrm {d}z}\bigg |_{z=0}, \quad \quad \lim _{z\rightarrow -\infty }n(z)=\kappa , \quad \quad \lim _{z\rightarrow -\infty }\phi (n)\frac{\mathrm {d}n}{\mathrm {d}z}=0. \end{aligned}$$

Following Fadai and Simpson (2020) and Fadai (2021) , we define

27 $$\begin{aligned} q(z) = \phi (n)\frac{\mathrm {d}n}{\mathrm {d}z}. \end{aligned}$$

Multiplying (25) by $\phi (n)$ and rewriting the conditions from (26) in terms of *q*(*z*), we obtain

28 $$\begin{aligned} n\phi (n)\frac{\mathrm {d}q}{\mathrm {d}z}=-q(c+q)-\phi (n)n(\kappa -n), \end{aligned}$$

29 $$\begin{aligned} n(0)=0, \quad \quad q(0)=-c, \quad \quad \lim _{z\rightarrow -\infty }n(z)=\kappa , \quad \quad \lim _{z\rightarrow -\infty } q(z)=0. \end{aligned}$$

Here, we note the second boundary condition from (26) transforms into the second of (29) because $\phi (0)=1.$ Dividing (28) by (27) we have

30 $$\begin{aligned} \frac{\mathrm {d}q}{\mathrm {d}n} = -\frac{q(c+q)+n(\kappa -n)\phi (n)}{qn}, \end{aligned}$$

31 $$\begin{aligned} q(\kappa )=0, \quad \quad q(0)=-c. \end{aligned}$$

Using the shooting method (presented in “Appendix B”) to find the heteroclinic connection *q*(*n*) that connects $(n,\,q)=(\kappa ,\,0)$ to $(0,\,-c)$, we can determine a numerical approximation of the wave speed in terms of the governing parameters $\kappa ,\,s,\,\mu$ and $\eta .$

**Fig. 2:**
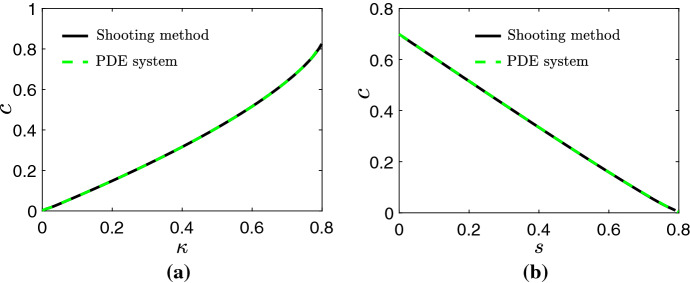
Numerical solutions for the wave speeds $c(\kappa )$ and *c*(*s*). The solid black and dashed green lines represent approximations sought by numerically solving (30) and (31) using a shooting method and the system from (18)–(20), respectively. Parameter values for **a** $\mu =\eta =L_0=1,\,s=0.2$ and $\omega =0.03.$ Parameter values for **b** $\mu =\eta =L_0=1,\,r_a/r_m=0.2$ and $\omega =0.03$ (Color figure online)

In Fig. 2a, b, the solid black line represents the relationships $c(\kappa )$ and *c*(*s*), respectively, when (30) and (31) is approximated by the shooting method. The dashed green line represents these wave speeds when obtained by numerically solving (18)–(20), and computing *c* by evaluating $\mathrm {d}L/\mathrm {d}t$ at large time. In view of the close agreement between these two approaches to computing *c*,  we henceforth concentrate on solutions provided by (30) and (31) for simplicity.

**Fig. 3:**
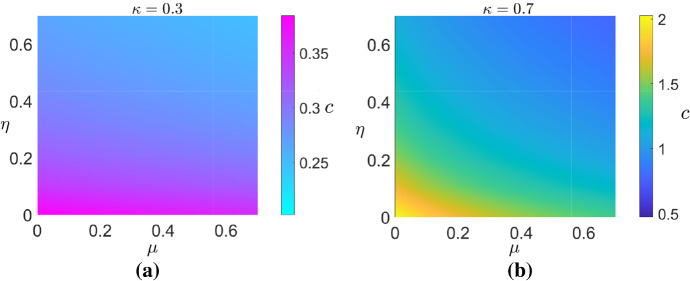
Numerical solutions for the wave speeds $c(\mu ,\,\eta )$ for $\kappa =0.3$ and $\kappa =0.7$ sought by numerically solving (30) and (31) using a shooting method. $s=0.2$ was used for both sub-figures. We note that the colour axes are different in each sub-figure (Color figure online)

The results presented in Fig. 2 suggest that larger $\kappa$ and smaller *s* increase the speed at which the tissue front grows, and since $\kappa =1-s-r_a/r_m,$ this further corresponds to minimising $r_a$ and maximising $r_m$ and the porosity of the scaffold. In Fig. 3a, b we present the wave speeds $c(\mu ,\,\eta )$ for $\kappa =0.3$ and $\kappa =0.7,$ respectively. These results suggest that, for a fixed value of $\kappa$, the wave speed is maximised when $\mu ,\,\eta \rightarrow 0.$ Physically, this corresponds to the case where the viscosity of the cells is much greater than the viscosity of the liquid, and where repulsive forces exerted due to cell–cell and cell–scaffold interactions at high cell volume fractions dominate inter-cellular forces that give rise to cell dispersal. Furthermore, Fig. 3a, b indicates that the dependence *c* has on $\mu$ is weaker for $\kappa =0.3$ than $\kappa =0.7.$ This suggests that for smaller $\kappa$, cell–cell and cell–scaffold interactions which can drive cellular motion are more prominent in controlling the wave speed than the cell and liquid viscosities. Additionally, and in agreement with Fig. 2a, Fig. 3 indicates that the wave speed increases as $\kappa$ increases.

## Asymptotic Analysis for $|\kappa |\ll 1$

In this section, we construct asymptotic solutions for $n(x,\,t)$ and *L*(*t*) for $|\kappa |\ll 1$ when $t\gg 1.$ Since scaffold porosity is a readily controllable parameter (in contrast to cell growth and death), the analysis in this section when $\kappa =1-s-r_a/r_m>0$ can be associated with the case in which the scaffold porosity is low and tissue growth is successful, so that $0<\kappa \ll 1.$

The numerical results in Fig. 1e indicate that $n\ll 1$ holds for $\kappa <0$ when $t\gg 1.$ Furthermore, given that $\max \{ n\}=\kappa$ when $\kappa >0,$ $n\ll 1$ and $t\gg 1$ is also expected when $0<\kappa \ll 1$ for sufficiently large time. We therefore have $\phi (n)\sim \phi (0)=1$ when $|\kappa |\ll 1$ and $t\gg 1$. To aid the subsequent asymptotic analysis, we introduce the variables

32 $$\begin{aligned} n=e^{\kappa t}N(x,\,T), \qquad T=\frac{e^{\kappa t} - 1}{\kappa }, \end{aligned}$$

which imply that (18) and (19) are simplified to

33 $$\begin{aligned}&\frac{\partial N}{\partial T} = \frac{\partial }{\partial x}\bigg (N\frac{\partial N}{\partial x}\bigg ) - N^2, \qquad 0<x<L(T), \end{aligned}$$

34 $$\begin{aligned}&\frac{\partial L}{\partial T} = -\frac{\partial N}{\partial x}(L,\,T). \end{aligned}$$

To analyse the behaviour of (33) and (34), we follow Newman (1980) and adopt the ansatz

35 $$\begin{aligned} N= A(T)-B(T)\cosh \bigg (\frac{ x}{\sqrt{2}}\bigg ), \end{aligned}$$

wherein $0<B<A\ll 1.$ Imposing $N(L,\,T)=0$ on (35), we obtain that

36 $$\begin{aligned} L(T) = \sqrt{2}\cosh ^{-1} \bigg (\frac{A}{B}\bigg ). \end{aligned}$$

In view of (35), (33) provides

37 $$\begin{aligned} \frac{\mathrm {d}A}{\mathrm {d}T} = -A^2-\frac{B^2}{2}, \qquad \qquad \frac{\mathrm {d}B}{\mathrm {d}T} = -\frac{3AB}{2}. \end{aligned}$$

In this section, initial conditions for *N* and *L* are chosen to satisfy (35) and (36) when $T=0$ – i.e., $A(0)=A_0,$ $B(0)=B_0$ and hence $L_0=\sqrt{2}\cosh ^{-1}(A_0/B_0).$ An implicit solution for *N* and *L* is found by computing *A*(*B*) from (37) and exploiting the relation from (36). Following Newman (1980), we have

38 $$\begin{aligned}&N(x,\,T) = \frac{\alpha }{2} {{\,\mathrm{csch}\,}}^3\bigg (\frac{L}{\sqrt{2}}\bigg )\bigg [ \cosh \bigg (\frac{L}{\sqrt{2}}\bigg )-\cosh \bigg (\frac{x}{\sqrt{2}}\bigg )\bigg ], \end{aligned}$$

39 $$\begin{aligned}&\sinh (\sqrt{2} L)-\sqrt{2}L=\alpha T + \beta , \end{aligned}$$

where $$\alpha = 2B_0^{-2}(A_0^2-B_0^2)^{3/2} $$ and $$\beta =\sinh (\sqrt{2} L_0)-\sqrt{2}L_0.$$

We now deduce the large-*T* behaviour of $N(x,\,T)$ and *L*(*T*),  from which the large-time behaviour of $n(x,\,t)$ and *L*(*t*) when $|\kappa |\ll 1$ can subsequently be determined. Guided by the numerical results from Fig. 1d, the evolution of *L* satisfies $L\gg L_0$ for sufficiently large *T*, so we have from (39) that

40 $$\begin{aligned} \frac{e^{\sqrt{2}L}}{2}-\sqrt{2}L=\alpha T+\mathcal {O}(1), \end{aligned}$$

which is then inverted to give

41 $$\begin{aligned} L(T) \sim \frac{\ln (2\alpha T)}{\sqrt{2}}\bigg (1+\frac{1}{\alpha T}\bigg ) \end{aligned}$$

for $T\gg 1$. Equation (38) and the leading-order term in the above expansion are used to find the following large-*T* approximation for *N*:

42 $$\begin{aligned} N(x,\,T) \sim T^{-1}-\sqrt{\frac{2}{\alpha }}\cosh \bigg ( \frac{x}{\sqrt{2}} \bigg )T^{-3/2}. \end{aligned}$$

We now exploit (42) and (41) to deduce the large-time behaviour of $n(x,\,t)$ and *L*(*t*) when $|\kappa |\ll 1.$

### Large-Time Behaviour of *n* and *L* When $|\kappa |\ll 1$

When $0<\kappa \ll 1$, then $n(x,\,t)$ takes the form of a travelling wave of constant speed and $L\gg L_0$ for $t\gg 1.$ In contrast, when $\kappa <0$, the numerical results in Fig. 1f suggest that $L\rightarrow L_{\infty }$ as $t\rightarrow \infty$ for some finite constant $L_{\infty }.$ In general, a large-time solution for *L* is unavailable given that $L_{\infty }\gg L_0$ does not necessarily hold when $t\gg 1;$ however, when $|\kappa |\ll 1,$ then $t=T$ to leading order at $t=\mathcal {O}(1),$ and *L* evolves according to (41) until $t=\mathcal {O}(1/|\kappa |)$. Since $L\gg L_0$ when $|\kappa |\ll 1$ and $t\gg 1$, (41) becomes

43 $$\begin{aligned} L \sim \frac{1}{\sqrt{2}}\ln \bigg (\frac{2\alpha (e^{\kappa t}-1)}{\kappa }\bigg ) \bigg (1+\frac{\kappa }{\alpha (e^{\kappa t}-1)}\bigg ) \end{aligned}$$

for $|\kappa |\ll 1$ and $t\gg 1.$ Equation (43) implies that $L \sim \kappa t/\sqrt{2}$ when $0<\kappa \ll 1$ and $t\gg 1.$ Therefore, travelling waves propagate with speed $c\sim \kappa /\sqrt{2}$ when $0<\kappa \ll 1,$ this being in agreement with the numerical results from Fig. 2a. For $0<\kappa \ll 1$, (43) implies that the growth of the tissue edge is logarithmic until $t=\mathcal {O}(1/\kappa )$ and linear thereafter. If tissue growth is successful, this suggests the formation of travelling waves with constant speed is delayed when the scaffold porosity is low. When $\kappa <0$, the exponential terms from (43) are negligible as $t\rightarrow \infty$ and we obtain

44 $$\begin{aligned} L_{\infty } \sim \frac{1}{\sqrt{2}}\ln \bigg (\frac{2\alpha }{|\kappa |}\bigg )\bigg (1+\frac{|\kappa |}{\alpha }\bigg ) \end{aligned}$$

when $\kappa <0$ and $|\kappa |\ll 1.$ The leading order logarithmic term in (43) and (38) is used to find the following large-time approximations for *n* when $|\kappa |\ll 1:$

45 $$\begin{aligned}&n \sim \kappa -\sqrt{\frac{2\kappa ^3}{\alpha }}\cosh \bigg (\frac{x}{\sqrt{2}}\bigg )e^{-\kappa t/2} \qquad \text {for} \qquad 0<\kappa \ll 1, \end{aligned}$$

46 $$ \begin{aligned}&n \sim \frac{|\kappa |e^{-|\kappa | t}}{1-e^{-|\kappa | t}}\bigg [1- \sqrt{\frac{2|\kappa |}{\alpha (1-e^{-|\kappa | t})}}\cosh \bigg ( \frac{x}{\sqrt{2}} \bigg )\bigg ] \qquad \text {for} \qquad \kappa <0 \,\,\,\, \& \,\,\,\,|\kappa |\ll 1. \end{aligned}$$

We note that (43) and (45) hold for $\kappa =\mathcal {O}(1)$ if $\mu \ll 1$ and $\eta \gg 1$ because $L\gg L_0$ and $\phi (n)\sim 1$ in this case. Furthermore, for $\kappa <0$ and $|\kappa |=\mathcal {O}(1),$ then (38) suggests that $n= \mathcal {O}( e^{\kappa t})$ at large time since $L_{\infty }=\mathcal {O}(L_0).$

**Fig. 4:**
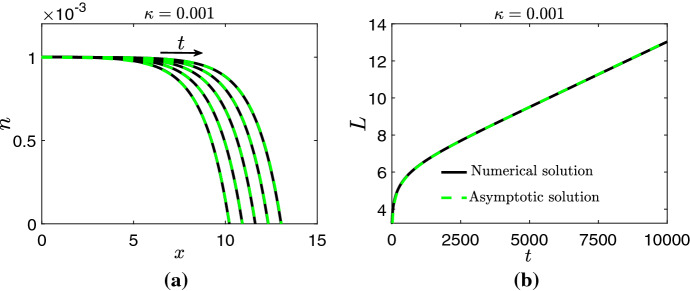
Numerical solution (solid black line) of the PDE system from (18)–(20) versus asymptotic solution (dashed green line) from (45) and (43) for *n* (**a**) and *L* (**b**) for $\kappa =0.001$. Solutions for *n* are presented on $t\in [6000,\,10,000]$ at intervals of 1000. Initial conditions for the numerical simulations were chosen to satisfy (35) and (36). Parameter values: $\mu =\eta =1,\,s=0.2,\,A_0=0.05$ and $B_0=0.01$ (Color figure online)

**Fig. 5:**
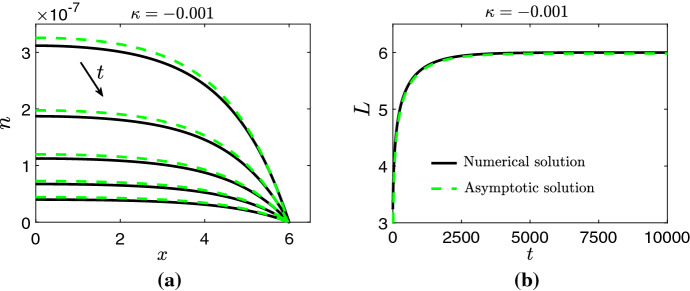
Numerical solution (solid black line) of the PDE system from (18)–(20) versus asymptotic solution (dashed green line) from (46) and (43) for *n* (**a**) and *L* (**b**) for $\kappa =-0.001$. Solutions for *n* are presented on $t\in [8000,\,10,000]$ at intervals of 500. Initial conditions for the numerical simulations were chosen to satisfy (35) and (36). Parameter values: $\mu =\eta =1,\,s=0.2,\,A_0=0.05$ and $B_0=0.01$ (Color figure online)

In Figs. 4 and 5, we compare the numerical solution for *n* and *L* when obtained by numerically solving the PDE system from (18)–(20) for $\kappa =0.001$ and $\kappa =-0.001$ against their respective asymptotic solutions from (45), (46) and (43). Overall, an excellent agreement between the numerical and asymptotic solutions is observed. The large-*T* behaviors of *N* and *L* characterised by (41) and (42), and hence the asymptotic approximations from this subsection, are only valid for initial conditions that satisfy (35) and (36). We now show that solutions of (33) and (34) converge to solutions similar to that of (41) and (42) for a wider class of initial data.

### Convergence of Asymptotic Solutions

Since the choice of initial cell distribution within the scaffold is likely to vary substantially in practice, it is important to determine the large-*T* behaviour of *N* and *L* for a wider class of initial data, such as those from (22). The asymptotic behaviour of (33) as $T\rightarrow \infty$ comprises an interior layer near the interface within which the similarity reduction $N \sim T^{-1}f(\theta )$ holds where $\theta =x-L$ and $L=b\ln (T),$ so that $f(\theta )$ satisfies

47 $$\begin{aligned} -b\frac{\mathrm {d}f}{\mathrm {d}\theta }=\frac{\mathrm {d}}{\mathrm {d}\theta }\bigg (f\frac{\mathrm {d}f}{\mathrm {d}\theta }\bigg )+f(1-f). \end{aligned}$$

Following familiar arguments to that of the porous-Fisher equation (Aaronson 1980; Murray 2002), although we emphasise that *N* is not a travelling wave of the usual form, the solution to (47) is given by

48 $$\begin{aligned} f = 1-e^{\theta /\sqrt{2}}, \quad \quad b=\frac{1}{\sqrt{2}}. \end{aligned}$$

For $$x=\mathcal {O}(1),$$ setting

49 $$\begin{aligned} N=T^{-1} + G(x,\,T) \end{aligned}$$

implies that

50 $$\begin{aligned} \frac{\partial G}{\partial T}=T^{-1}\frac{\partial ^2G}{\partial x^2}-2T^{-1}G, \end{aligned}$$

the solution to which that matches into the exponential terms in (48), and hence the corresponding term in the interior layer, is given by

51 $$\begin{aligned} G = -m\cosh \bigg ( \frac{x}{\sqrt{2}}\bigg )T^{-3/2} \end{aligned}$$

for some unknown constant *m*, and this dominates the asymptotic behaviour of (50) as $T\rightarrow \infty$. Therefore, (49) becomes

52 $$\begin{aligned} N\sim T^{-1}-m\cosh \bigg ( \frac{x}{\sqrt{2}}\bigg )T^{-3/2} \end{aligned}$$

where *m* depends on the initial data. Since $N(L,\,t)=0,$ (52) implies that

53 $$\begin{aligned} L \sim \sqrt{2}\cosh ^{-1}\bigg (\frac{\sqrt{T}}{m}\bigg ) \sim \frac{1}{\sqrt{2}}\ln \bigg (\frac{4T}{m^2}\bigg ) \end{aligned}$$

for $T\gg 1$. By comparing (52) and (42), we see that the asymptotic structure is retained despite the initial cell distribution for large *T*. In addition, (53) suggests that the choice of initial cell distribution does not affect the speed at which the tissue edge moves for large *T*, but does affect the position of the tissue boundary. We note that if $N(x,\,0)$ is chosen to satisfy (35), then (42) indicates that $m=\sqrt{2/\alpha }.$

**Fig. 6:**
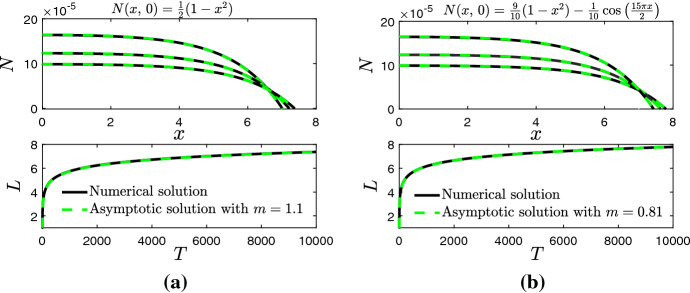
Numerical solution (solid black line) of the PDE system from (33) and (34) versus asymptotic solution (dashed red line) from (52) and (53) for *n* and *L* for different initial conditions $N(x,\,0)$ and $L_0=1$. Solutions for *n* are presented on $T\in [6000,\,10,000]$ at intervals of 1000 (Color figure online)

In Fig. 6, we compare the numerical solutions for *N* and *L* when obtain by numerically solving the PDE system from (33) and (34) against the asymptotic solutions from (52) and (53) for two choices of $N(x,\,0)$ and $L_0=1$. The value of $L_0$ was found by solving $N(L_0,\,0)=0.$ For both $N(x,\,0)$, we are able to choose an *m* that provides excellent agreement between the numerical and asymptotic solutions. We note that the large-time behaviour for $n(x,\,t)$ and *L*(*t*) when $\kappa =0$ can be extracted directly from (52) and (53) given that $N=n$ when $\kappa =0$ and $\lim _{\kappa \rightarrow 0}T=t.$ This justifies the numerical results observed in Fig. 1c, d.

## Conclusions

In this paper, we present a multiphase model to represent the development of tissue in vitro within a porous tissue engineering scaffold. We consider a tissue cell phase, extra-cellular liquid phase and a scaffold phase and adopt Darcy’s law to relate the velocity of the cell and liquid phases to their respective pressures. The model includes mechanisms to represent cell growth and death, and pressures that arise from cell–cell and cell–scaffold interactions. We employ a moving boundary, $x=L(t),$ to track the speed at which the tissue edge propagates through the scaffold. We reduce the model to a nonlinear reaction–diffusion equation for the cell volume fraction, $n(x,\,t)$, and a moving boundary condition for the tissue edge. The diffusivity of the reaction–diffusion equation is dependent on the cell and scaffold volume fractions; cell and liquid viscosities, and pressures that arise from cell–cell and cell–scaffold interactions. Non-dimensionalisation of the model shows that the tissue boundary position increases with the scaffold permeability and exposes important dimensionless groupings. One such grouping, $\kappa ,$ that describes the difference between the scaffold porosity and the ratio between the cell death and growth rates is of crucial importance to the qualitative features of the cell phase evolution. The model admits three regimes for the evolution of the cell volume fraction and the moving boundary, based on the sign of $\kappa$. Employing travelling-wave and asymptotic analysis, we characterise these regimes in terms of $\kappa$ and parameters related to cellular motion.

The case in which $\kappa >0$ corresponds to the successful growth of tissue, which suggests that tissue engineers should ensure that the porosity of the scaffold is at least larger than the ratio between the rate of cell death and growth. For $\kappa >0,$ we show that the cell volume fraction, $n(x,\,t),$ spreads through the scaffold as a semi-infinite travelling wave with constant speed, emerging from the steady state $n=\kappa .$ Employing travelling-wave analysis, we accurately compute the wave speed (i.e., the speed at which the tissue edge moves through the scaffold) as a function of the governing parameters. We find that the wave speed is greatest when the rate of apoptosis is negligible in comparison with that of mitosis, the viscosity of the cells is much greater than the viscosity of the liquid, and when repulsive forces exerted due to cell–cell and cell–scaffold interactions at high cell volume fractions dominate inter-cellular forces that give rise to cell dispersal. We also find that the wave speed increases as the scaffold porosity increases; however, we note that the cells will require a sufficient amount of scaffold on which to attach, so an upper bound on the porosity is to be expected. Furthermore, we deduce that for smaller values of $\kappa ,$ and hence scaffolds with small porosities, cell–cell and cell–scaffold interactions which can drive cellular motion are more prominent in controlling the wave speed than the cell and liquid viscosities.

For $|\kappa |\ll 1,$ we employ asymptotic analysis to find explicit solutions for *n* and *L*. Since scaffold porosity is a readily controllable parameter (in contrast to cell growth and death), the analysis in this section when $\kappa =1-s-r_a/r_m>0$ can be associated with the case in which the scaffold porosity is low and tissue growth is successful, so that $0<\kappa \ll 1.$ When $|\kappa |\ll 1,$ the growth of the tissue edge is logarithmic until $t=\mathcal {O}(1/\kappa )$ and linear thereafter, thus suggesting the formation of travelling waves with constant speed is delayed as $\kappa \rightarrow 0^+,$ and hence when the scaffold porosity is low. For $\kappa <0,$ we deduce that the cell volume fraction decays exponentially with rate $\kappa$ at large time, with the moving boundary tending towards a steady state. For $\kappa <0$ and $|\kappa |\ll 1$, the evolution of the *L* is shown to be logarithmic until $t=\mathcal {O}(1/\kappa )$ and approaches a steady state thereafter, the value of which is found explicitly and related to $\kappa$ and the initial conditions employed in the model. For $|\kappa |\ll 1,$ we also demonstrated that the choice of initial cell distribution does not affect the eventual distribution of cells within the scaffold, nor the speed at which the tissue edge moves, but does affect the position of the tissue boundary.

For a functional tissue construct to develop within a scaffold, cells must be exposed to the correct environment and stimulated with growth factors such as oxygen. There must also be a sufficient amount of scaffold on which the cells can adhere. Whilst key features of tissue growth such as cell mitosis, apoptosis and motion are included in this paper, concepts such as environmental pressures, cellular adhesion, and nutrient supply have not been considered. Therefore, following Lemon and King (2007a), a natural extension of this work would include examining the influence that nutrient limitation has on cell growth. We leave these extensions for future consideration.

## Data Availability

The MATLAB codes employed to generate the results presented in all figures can be found at: https://github.com/JacobJepson/scaffoldcodes_2021.

## A Linear Stability Analysis

A linear stability analysis around the steady states of (18), $n_{\infty }=0,\,\kappa ,$ provides insight into the dependence of the model behaviour on $\kappa$ and $\phi (n)$. Neglecting the influence of the moving boundary condition on the stability of (18), we linearise on a semi-infinite domain. We substitute $n = n_{\infty } +\varepsilon \exp (i\gamma x+\lambda t)$ into (18) for a perturbation wave number $\gamma$ and growth rate $\lambda$ where $\varepsilon \ll 1$. Considering terms of $\mathcal {O}(\varepsilon )$ only, the growth rate for perturbations of wave length $2\pi /\gamma$ is

54 $$\begin{aligned} \lambda = -n_{\infty }\big [\gamma ^2\phi (n_{\infty })+2\big ]+\kappa . \end{aligned}$$

Since $\phi (n)$ is assumed to be positive for any *n*, the steady state $n_{\infty }=\kappa$ is stable for all $\kappa >0$. For $n_{\infty }=0,$ we have $\lambda =\kappa$ which indicates stability when $\kappa <0$.

## B The Shooting Method

In this section, we formulate a numerical shooting method to find the wave speed *c*,  as stated in Sect. 4. To do this, we send trajectories of *q*(*n*) from $(\kappa ,\,0)$ to find a trajectory that connects to $(0,\,-c).$ Computationally, it is more straightforward to shoot trajectories forwards as opposed to backwards, and we hence introduce the change of variable $X=\kappa -n$ and obtain

55 $$\begin{aligned} \frac{\mathrm {d}q}{\mathrm {d}X} = \frac{q(c+q)+X(\kappa -X)\phi (\kappa -X)}{q(\kappa -X)}, \end{aligned}$$

56 $$\begin{aligned} q(0)=0, \quad \quad q(\kappa )=-c. \end{aligned}$$

Noting that the end points are computationally singular, we use $q(\zeta )=-\zeta$ and $q(\kappa -\zeta )=-c$ where $0<\zeta \ll 1$ is some user-defined tolerance. We employ the discrepancy function

57 $$\begin{aligned} E(\overline{c}) = q_{\,\overline{c}\,}(\kappa -\zeta ) + \overline{c}, \end{aligned}$$

where $q_{\,\overline{c}\,}(\kappa -\zeta )$ is the solution to (55) evaluated at $X=\kappa -\zeta$ for a trial wave speed $\overline{c}.$ Equation (55) is numerically integrated with ode23s in MATLAB with the initial condition $q(\zeta )=-\zeta .$ Using fzero in MATLAB to find the zero of $E(\overline{c}),$ the wave speed *c* and the corresponding heteroclinic trajectory *q*(*X*) is determined.
